# Pedagogical Leaders and the Teaching—Learning Processes in COVID-19 Times

**DOI:** 10.3390/ijerph18157731

**Published:** 2021-07-21

**Authors:** Emilio Álvarez-Arregui, Eufrasio Pérez-Navío, Raúl González-Fernández, Alejandro Rodríguez-Martín

**Affiliations:** 1 Department of Education Sciences, University of Oviedo, 33005 Oviedo, Spain; alvarezemilio@uniovi.es; 2 Department of Pedagogy, University of Jaén, 23071 Jaén, Spain; epnavio@ujaen.es; 3 Department of Didactics, School Organization and Special Didactics, National Distance Education University (UNED), 28040 Madrid, Spain; raulgonzalez@edu.uned.es

**Keywords:** pedagogical leaders, COVID-19, teaching–learning planning, teacher training, competencies

## Abstract

This study aimed to identify the main decisions made by pedagogical leaders, comprising institutional management teams, heads of departments, and teachers in general, in order to improve teaching–learning processes and promote the comprehensive education of students—in particular, secondary school students during the pandemic period, located in the regions of Andalusia and Madrid (Spain). An integrated mixed methodology was applied, composed of the contributions of discussion groups and content analysis of the corresponding open questions presented in each of the constitutive dimensions of the questionnaire. Such analyses were expanded by applying an ad-hoc designed questionnaire, which indicates the main actions developed by leaders and their implications in the educational community, when working on communication facilitation processes, using program planning through the support of technologies and decision making regarding the training of teachers, didactic resources, and the emergence of competences to be trained by the leaders participating in the research. The results highlight the dedication, commitment, and implication of pedagogical leaders during the COVID-19 period.

## 1. Introduction

The questions raised by pedagogical leaders regarding the development of teaching in pandemic times need be answered, and an increased awareness of the many challenges lying ahead due to COVID-19 needs to be fostered. Harris and Jones [1] and Labelle and Jacquin [2] have underlined different complex situations that were unheard of until this technologically innovative century. The complicated and complex COVID-19 crisis has highlighted many problems in education, which cannot be resolved on their own [3]. The emergence of new queries and personalized attention demands, the necessary implication of institutions and ecosystems, and the globalization of teaching trends all require particular insights.

Medina, De la Herrán, and Domínguez [4] have advocated for a reflection and decision-making process enabling improved understanding, humanization, and adequate answers to the true challenging issues of the pandemic: health, empathy, understanding, cooperation, creation of an emotion-based culture, development of rational thought, and so on. Such issues need be embodied by different pedagogical authorities and reverted using innovative adaptations and creative answers in order to face these uncertain times.

The fundamental objective of the present research was to demonstrate the impact of crisis situations on educative systems and pedagogical leaders. They become more conscious of their professional importance in society in order to convert education into an integral training activity and facilitate the preparation of future generations to overcome uncertain times such as those provoked by the COVID-19 pandemic. Such reflection is carried out to help unveil the most valued competences (i.e., developed in pandemic times) that generate an adapted and creative learning environment.

Gento, González-Fernández, and Silfa [5] have provided evidence of the importance of pedagogical leadership and its affective dimension for teachers themselves, together with the participative and affective dimensions. Their results indicated an existing relationship between pedagogical leadership and the rapprochement or recognition of the inherent dignity of every member within these educative organizations.

Hargreaves and Shirley [6] have shown that pedagogical leadership is a key element inside educative institutions, where teachers and their educative roles play the most important part. Teachers undertake leadership when focusing on understanding the learning process of their students, when improving cooperation with their colleagues, or performing reflective actions to improve didactic interactions [7].

## 2. Background

The pedagogical leader model has been designed and based on diverse characterizations and profiles that are adapted to the context, typology, and projection of such leadership, in order to provide for the integral improvement of schools. Progin Perrenoud [8], following the research line initiated by Garant and Letor [9], considered leadership not as a mere personal characteristic but, rather, as a dynamic process of social influence, where a person has a ‘deliberate influence on others to help develop activities and promote adequate relationships between all members within an organization’.

Contreras [10] understood pedagogical leadership as a synthesis of distributed and participative leadership and pedagogical professional development. Day [11] (p. 139) defined school leadership as the teaching performance, where the main nuclear components are ‘professional autonomy, professional capital, teacher commitment, well-being and expertise’. Accordingly, Labelle and Jacquin [2] (p. 178) represented it as ‘the principal transformational leadership role within professional learning communities [...] an overview of different leadership styles to provide a frame of reference for integrating the idea of transformational leadership into a broader national network’.

Being a pedagogical leader means that the teachers in charge of the main decisions (school principal/head of subject) need to help improve the teaching–learning process, provide the basis for such a transformation within the educational community, and optimize the results and training of students. Tschannen-Moran [12] and Bullough [13] identified some defining aspects of an efficient leader: the establishment of set values, educational management, improvement of teaching–learning conditions, new design of organizations and educational programs, positive relationships with the entire educational community, optimism, moral integrity, professional belonging feelings, autonomy and teaching expertise; these aspects have also been defined by Leithwood et al. [14].

Educational institutions are quite different from one another, and may also take on complex pedagogical functions. To understand their function in pandemic times, researchers need to use different models to reflect upon and obtain an integral vision that is to be shared by all members of the educational community [15]. For González-Fernández et al. [16], building up a pedagogical leadership model and providing for some valuable affective dimension is important. Many authors have provided evidence for the high relevance of such a dimension. The pedagogical leadership of teachers must also complement the participative and training dimensions with some professional practice, based on the understanding and acknowledgement of the role played by every member of the educational community, together with their particular needs and priorities [17].

Teachers assume leadership when they provide for a concrete understanding of institutional dimensions; they focus on understanding the student training process, on cooperation between colleagues, and on finding reflexive orientations to improve the educational actions and the didactic interaction [7,15]. The full evaluation of all implications of protagonists inside schools is important to improve the relationships between all and to develop institutional structures that are sufficiently (culturally) mature and globally prepared to provide new ideas to teachers, students, and relatives and, finally, foster understanding within these training communities [18,19].

The assertion of this leadership, as a dual vision of identification with the teaching activity and its implication within education itself, has been reviewed by Leithwood [20] and further amplified by the vivid intensity of the uncertainty experienced during the year 2020. Teachers are now responsible for more decisions, and the identity or style of every leader is patent during the teaching activity, due to the associated complex contexts [21]. Clapp-Smith et al. [22] have suggested training leaders in four main identities: ‘meaning, strength components, levels (personal, relational and collective) and integration. The creation of a narrative and personal reflection and group discussion on the leader identity’. Such a leadership process needs be developed in an intensely cooperative environment [23] as, in these contexts, educators tend to show self-efficacy, job satisfaction constructivist beliefs about teaching, and learning and use of teaching strategies [24].

When lived closely by all co-partners within institutions, network leadership achieves high levels of interaction; this is amplified by the secondary effects and quality of all messages experienced [25]. Leithwood [20] analyzed the ways that leadership is performed in networks, with a particular interest for network leadership, network health, network structure, and network connectivity, which are considered as fundamental components of informal and experience-based training, characterized by the use and sharing of training contents online, together with a high interdependency of participants during this process. Projects developed through networks provide useful elements to understand the complex and uncertain characteristics of new horizons and situations, where leaders interact and discover creative solutions to answer the issues raised during the pandemic [1].

During the pandemic, uncertain and complex situations have been experienced by educational institutions, testing the necessary competencies and training-transformational values—consolidated by school principals and used to achieve integral education—and the commitment and cooperative environment of the whole educative community [26]. Lamm et al. [27] underlined the importance of such transformational leadership, where major support is given to the organization members and where an increased commitment of participants is generated to meet the pre-set objectives. Vermeulen, Kreijns, and Evers [28] helped to facilitate progress in the understanding of the new significance of these complex issues faced by institutional cultures; this research line has highlighted the importance of the transformational leader in fully potentiating and developing a real educational interaction during highly uncertain times, such as those experienced in 2020.

Another major issue is that of distributed leadership. Defining it as merely delegating tasks to other colleagues is very reductive, as it also implies the designation and implication of teachers and other educational community participants, in order to assume new roles and styles of shared responsibility [29,30,31], while learning to make decisions in difficult times. In this respect, Harris and Jones [1] (p. 242) stated: ‘This year, COVID-19 has redefined learning as a remote, screen-based activity, limiting most learners to on-line teacher support and, at the moment, schools have faced considerable challenges: social distancing, intensive cleaning and the careful orchestration or all movements.’ They advocated for some new priorities: learning to learn, learning to connect, understanding of the crisis process and assessment change, the importance of communities to provide support and assume distributed leadership, and relationships based on empathy.

Diez-Gutiérrez and Gajardo-Espinoza [32] have provided evidence of the digital, social, and educative divide between students and families facing educational challenges during the pandemic in Spain. ICTs have proven to be a bad alternative to presential education and relationships, though they ease the teaching–learning process at a distance. The ICT solution requires specific adaptation of education programs and the selection of nuclear contents, together with the set-up of tests and support means to help facilitate understanding of the subject by students. ICT has revolutionized communicative, training, and work processes [33]. This is particularly challenging for pedagogical leaders due to the urgency and immediate decisions that are to be made and, due to the scarcity of means, the lack of digital expertise, the missing didactic interaction and empathy, and the lack of registered previous experiences [34,35]. Vertical and shared leadership within professional and learning communities is important [36], as teachers must co-operate to design educative materials [37]. Accordingly, Dirani et al. [38] have shown that leaders must improve the decision-making process, improve self-esteem and confidence, and maintain close cooperation between all institutional members. These authors have provided a classification of the best leadership practices, establishing a difference between the actions performed in normal and crisis times (Table 1).

In times of crisis, the main leadership competencies are synthesized, and the action style becomes based on the decision-making process and the reciprocation of confidence in order to overcome the crisis responsibly and collaboratively [39].

The complexity of situations experienced during confinement has led leaders to react with some productive teaching and learning processes, using technological means to interact with colleagues, families, and students, while looking for distributed, collaborative, and networking leadership [40,41]. Uncertainties provide evidence that leaders are assuming new ways of supporting teachers and generating creative lines to provide opportunities and develop future educational methods [42,43].

In uncertain times, the diverse functions performed by leaders show that every person needs some support from other colleagues. The leaders themselves need support [44] and require essential competencies commonly identified as human competencies (empathy, emotional closeness, and understanding), management competencies (optimization of resources, time planning, efficient decision making, and function distribution), and technical competencies (pedagogical expertise, didactic model generation, innovative culture creation, information organization, and ICTs expertise). García-Cobo et al. [45] have shown that pedagogical leaders need new skills to assume the complexity of activities: ‘clear timetables, mix planned activities, teacher skills and technologies, connections with parents, psychological support to communities, and communication to many platforms and social nets’.

The diversity of assumed functions and the transformations performed to reorient the training process provide for a new frame and program design that capacitate leaders to be aware of the new challenges lying ahead [46,47]. There are also limits to the capacity of leaders regarding the professional development framework and the emergence of national standards, in terms of competences, knowledge, and behaviors, which are most needed for those working in different services.

In uncertain times, coherent competences are needed to face the challenges that arise. During pandemics, competences considered as essential include communication, digital knowledge, empathy, innovation, emotional stability, didactic resilience, and interaction [48]. These competences then surge in providing decision-making combinations and styles, empowering leaders to assume their responsibility and provide for a global commitment, based on some balanced emotions and intense empathy. Hulpia and Devos [30] underlined the major qualities of shared leadership and of school organization, social interaction, team cooperation, and participative decisions, a leadership vision also shared by other authors such as Bouwmans et al. [49].

Pedagogical leaders (both teachers and principals) have made prudent and efficient decisions in order to improve their support to students discovering new ICT resources, accepting the complex tasks with determination and a notable moral role [50]. Due to the huge and irreversible situation lived inside schools and the main educational systems in general, new and complex responsibilities have emerged.

These have helped to identify the needs of students, families, educative communities, and the demands of administrations themselves. The whole picture will allow for mapping of the future challenges to be faced by all and, most particularly, those faced by pedagogical leaders performing new tasks of high personal and professional impact [51].

## 3. Materials and Methods

### 3.1. Objectives

The main objectives of this research were:

- To determine which decisions were made by pedagogical leaders to improve the training practices and the teaching–learning process and provide an integral education of all students during the COVID-19 pandemic.

Our specific objectives were:

- To assess the tasks performed by pedagogical leaders to improve educational processes during the pandemic period (COVID-19);
- To analyze the processes of planning and reprogramming of teaching, as well as the adaptation/training of teachers;
- To understand the educational interaction generated during the pandemic period in educational institutions and cyber-classrooms;
- To identify the competences that pedagogical leaders must develop in order to adequately perform educational processes in institutions.

### 3.2. Methods

The methodology used was based on a mixed vision. It encompasses the lines of Tashakkori and Teddlie [52], both evidencing the richness and relevance of approaches and data using experimental methods and questionnaires. Furthermore, Levitt et al. [53] advocated for intensification of the narrative, the understanding, and the integral vision of the plurality of expressions, considering the complexity of training situations and their ever-changing nature. The large panel of issues presented by focus groups and the complementary data collected by the questionnaires helped us to obtain an improved understanding of the significance of human relationships, as experienced during the pandemic by both teachers and school directors.

### 3.3. Instruments

An ad-hoc questionnaire was designed, following the model provided by Marqués [54]. Some constitutive dimensions related to the COVID-19 challenge, the teaching–learning process and competency planning, and the characteristics of leadership performance were included. Other different issues were re-designed in some dimensions, after questionnaire validation was carried out, through consulting 20 experts. The items were contrasted by the researchers themselves. The reliability was assessed using the SPSS 24 software, resulting in a Cronbach’s alpha of 0.960.

The questionnaire was divided into 10 dimensions: Identification (first one), and the following correspond to the essential aspects of pedagogical leaders during the COVID-19 crisis and its impact on their work. All questions were adjusted to each dimension and fulfilled internal harmony, parsimony, and homogenization criteria. Open-ended questions were also included, with at least one for every dimension. A total of 75 items were presented using a Likert scale (1, totally disagree; 6, totally agree).

The configuration of the set of questions in the questionnaire followed the order established in the 10 defined dimensions, where each of the constitutive dimensions culminated with at least one open question (as with Questions 10, 19, 20, 34, 36, 49–53, 59, 60, 67 and 73). Questions 45–48 were open multiple-choice questions, which were considered in the qualitative analysis carried out.

Four focus groups were set, with a total of 28 participants. They shared their own experiences and educative implications during the pandemic. The items developed by the focus groups were selected after reviewing the answers collected in the open-ended questions of the questionnaire.

### 3.4. Sample

The sample selected (N = 100) was incidental and fulfilled two criteria; it included teachers and leaders of educational institutions based in Spain, with a particular emphasis on the Andalusian and Madrid Regions. Teachers with previous teaching experience represented Secondary Education (61.7%), University teachers (25.5%), and some teachers in Primary and Pre-school Education (12.8%). The teaching experience ranged from 1–10 years (14.8%), while those with from 11 to 19 and with more than 20 years of teaching experience having the same percentage (42.6%). More women (76%) were represented in the present research, in coherence with the tendency observed in the overall education system (most particularly, in compulsory school stages).

Through repeated visits to the corresponding centers at a time of maximum complexity, we reached 100 responses to the questionnaire. We utilized repeated insistence and collaboration of supervisors in situations of maximum uncertainty in order to obtain the answers obtained, and our gratitude was expressed. Numerous insistences on situations of great complexity made it possible to obtain the 100 questionnaires answered. The set of constituent elements of the questionnaire numbered 75.

### 3.5. Data Analysis Process

Quantitative data analysis was carried out using the SPSS 24 software in order to extract statistics on central tendencies and descriptive data for every dimension and item (see Appendix A). The R software was used to contrast the sample adequacy and to carry out the Bartlett test in order to compare the dependence of variances—both fundamental bases to apply further factorial analysis (FA).

For the qualitative analysis, all debates were transcribed and enriched by a further analysis and dialogue with all participants, expressed with a high dose of empathy, educative characterization, and interactive harmony. For the focus groups and open-ended questions, content analysis based on grounded theory was applied, allowing us to identify the main codes and an additional categorization of data using the inference [55].

In the first place, researchers read the written material several times, acquiring a deep level of familiarity with it. Then, they worked independently, dividing the text into smaller sentences and assigning a specific code to each of them. In a following phase, researchers reviewed and discussed individual interpretations, verifying whether the identified codes were the same and eliminating possible redundancies. Similar codes were discussed and clustered in order to reduce them to the smallest number possible. Finally, the data were organized in semantic networks using the Atlas.ti 8.0 program. For the focus groups and open questions, content analysis based on grounded theory was applied, which allowed for the identification of the main codes and an additional categorization of the data through inference [55]. First, the researchers read the written material several times, gaining a deep level of familiarity with it. Then, they worked independently, dividing the text into smaller sentences and assigning a specific code to each of them. In a next phase, the researchers reviewed and discussed the individual interpretations, verifying whether the identified codes were the same and eliminating possible redundancies. Similar codes were discussed and grouped together in order to reduce them to the smallest number possible. Finally, the data were organized in semantic networks using the Atlas.ti 8.0 program. This content analysis complied with the standard procedures proposed by Huber [56] and Saldaña [57], and it incorporated our own effective methodology that has already been evaluated in previous studies [58,59]. In Table 2, a synthesis of the referents used is provided.

## 4. Results

### 4.1. Descriptive Analysis

All the dimensions contained in the questionnaire reached high values in the 1–6 point scale. The lowest averages obtained corresponded to the dimensions VII ($\overset{-}{X}$ = 4.57) and III ($\overset{-}{X}$ = 4.97), and they represented values that are higher than the possible average of means (see Table 2). The other dimensions were distributed as follows: Most valued was X ($\overset{-}{X}$ = 5.44), followed by IV ($\overset{-}{X}$ = 5.20), V ($\overset{-}{X}$ = 5.19), VI ($\overset{-}{X}$ = 5.06), and II ($\overset{-}{X}$ = 5.09), then by VIII ($\overset{-}{X}$ = 5.02) and IX ($\overset{-}{X}$ = 5.01); see Table 3.

A descriptive analysis of the questions indicated that only three questions reached an average of $\overset{-}{X}$ = 4.45: ‘training families’, ‘performing on-line tasks’, and ‘promoting jointly the tasks to be done’. Similar values were reached by the ‘communication planning’, ‘individual training activities’, ‘social media use’, and ‘students’ coaching’ questions. Other questions obtained mean values between 4.70 and 5.71, corresponding to human competences with high values (see Appendix A).

### 4.2. Factorial Analysis

The factorial analysis applied considered the correlative value between variables for every dimension. Selection criteria were those reaching a correlation higher than 0.4, as follows: For the first factor, we ignored 9, 11, 12, 39, 41, and 42; for the second factor, 45 and 49–52; and, for the third factor, only 75 was ignored. The results presented a value of 0.76 for KM0, indicating that the correlation between variables was high. Bartlett’s test reached 23588.36 (*p* = 0.00), indicating the homogeneity of variances, which confirmed the possibility to perform the factorial analysis. This FA provided, as a result, three factors, obtained by transforming obliquely:

- F1: Teaching and learning process planning and development (digital training/ICT, communication, and program adaptations).
- F2. Training activities and teacher professional training.
- F3: Pedagogical leader competencies

All the tests used (see Table 4 and Table 5) adaptation and factor correlation, together with the Fit, based upon off diagonal values (0.98), indicating the quality of the factorial solution.

For the variables whose weight in the corresponding factor obtained a value lower than 0.4, it was understood that they were not appropriate to incorporate into the global explanation due to the insufficient values obtained.

#### 4.2.1. Factors 1 and 2

The factorial analysis (see Table 6) represented a balanced structure with an important presence of the first factor inside the variance group, where all dimensions were represented: from the digital competence of students and families to the planning/program of the teaching–learning process, as well as educational interactions. This factor synthesizes the tasks and challenges experienced and worked on by pedagogical leaders in order to consolidate the training activities and promote an integral education for all students during the COVID-19 pandemic.

The enlarged analysis coincided with the harmonization of five dimensions and a high value for each of the variances (from 0.400 to 0.884), which accounted for an elevated coherence between the findings, meeting the average obtained by the descriptive analysis in their respective dimensions, where the lowest average ($\overset{-}{X}$ = 4.97) corresponded to Communication Planning and the highest to ICT resources and Program adaptations ($\overset{-}{X}$ = 5.19 and $\overset{-}{X}$ = 5.20, respectively; see Table 3).

The second factor integrated dimensions VII and IX and included eight essential questions, answering the training activities of students and the professional development of teachers.

This factor explains the main aspects to prepare students and teachers during this crisis period. Dimension VII ($\overset{-}{X}$ = 4.57) obtained the lowest value within the group, though the variance weight for this variable was high, confirming the high value and pertinence of ICT support, with a high weight (0.810) for this factor.

#### 4.2.2. Factor 3

The third factor (see Table 7) comprised the items dedicated to the overall competencies and confirmed the harmony and correspondence between dimension X and the corresponding factor. This dimension obtained the highest value in the descriptive analysis ($\overset{-}{X}$ = 5.44), as confirmed by the weights corresponding to the planning and management competencies (0.941 and 0.959, respectively).

The answers to the questionnaire by the pedagogical leaders coincided with those emerging from the qualitative analysis.

Consistent with factors 1 and 2 from FA, the results emerging from the analysis of open-ended questions included in the questionnaires and focus groups indicated the main challenges faced by educational leaders during the COVID-19 pandemic (see Table 8).

The training online (TON) metacode (see Table 8) produced 578 significant segments (sentences). The segments were codified (grouped) into 73 categories, which were classified into five catalogues, referring to perceived context (PC-10), used tools (UT 10), actions developed (AD-15), needs detected (ND-22), and training proposals (TP-16).

From a broad perspective (dimension), we can affirm that teachers generally associated the perceived context (PC) in online training (TON) and considered it to be coherent in terms of the health protocols that have been implemented (CHP), although there had been too much external dependence in its application, which limited its effectiveness (DIP) as it has given too much redundant information (RCO). Individual technological initiatives (ITI) have prevailed over collective ones, which generated loneliness in teachers (STE). In any case, it was widely indicated that the political protocols (PIP) have been insufficient in terms of the methodologies to be used (AMD); there have not been enough resources (MER) to carry out quality teaching practices. The legal framework to work with these protocols were found to be obsolete (OLF) in the times of COVID-19.

The tools used (UT) in online training (TON), in addition to individual or family computers, were mainly: the internet (IN), web pages (WP), email (EM), mobile phone (MP), distribution lists (DL), google meets (GM), google classroom (GC), and wasp (WA).

The actions developed (AD) in online training (TON) were mainly related to reading information (RI), searching for information on web pages (SI), completing information in texts already prepared (CI), and analyzing the information (AI). The teachers considered that there had been an increase in the time of dedication, especially in the management of administrative tasks in the network (EM). To a lesser extent, they worked as a team (TE) and in a collaborative network (NW) as they have not had sufficient training (DT) in the didactic use of digital tools (TM), in planning (PT), or in student orientation (SA) with the new teaching model (TON).

The needs that were detected (ND) were generally associated with the moment of change that we are experiencing as a society, which must be reflected in teaching (CCT), in the management of educational centers (CCD), and in educational administration (CCA). The teachers considered that there must be greater involvement of politicians (PIE), people who manage the centers (PIED), the administration (IAE), and companies linked to education (EIB). The teachers indicated the importance of the capacity to learn to learn (CLL), digital curricular adaptations (CCA), planning (CPL), institutional and teacher leadership (CLI), resilience (RECA), empathy having not been sufficiently developed (EMCA), and motivation (MOCA). The teachers insisted that they have not had enough advice (CDA), that the work platforms have not always worked correctly (EDP), and that regulations were not adapted to the emerging needs (AR).

The proposals they made for improvement were mainly linked to digital training (PT). Such training was mainly related to institutional management (DMT-M) and leadership (ET-LI), but also involved the digital administrative management that teachers have to develop (DMT-T), as well as learning to improve communication systems (ET-C). Teachers are aware that they need more training to use the platforms more fluidly in their professional relationships (DET-DO), in the relationships they establish with families (DET-F), and in those they develop with students (DET-A). The teachers considered that they need to be trained in the new hybrid teaching–learning environments, in terms of planning (ET-PL), evaluation (ET-EV), learning to learn (ET-LL), learning to undertake (ET-EL), and multidisciplinary work (ET-MW). With this training, they will be able to improve personal and institutional resilience capacity (ET-R), motivation of the people involved (ET-MO), and empathy (ET-EM).

Examples of these arguments can be found in the following meaning segments, which are grouped based on the dimensions and indications provided in the tables (all translations are the authors’ own):

Context perceived (PC):

“*In these months of work in a pandemic, we have had many professional experiences because new proposals have been designed and other resources have been used; the problem is that, on many occasions, I have felt alone to face these tasks and it generated anxiety” (FG.003.W.1.TON.PC.ITI).*

*“In our school, teachers have greatly improved their digital skills in these months, but we have not always felt sufficiently supported by politicians and the center’s management” (FG.002.M.2.* *TON.PC.PIP).*

*“The main problem was that the teachers felt overwhelmed and the leaders, at times, could not balance the workflow with the needs […] although the problem now seems solved” (QO.033.W.2.TON.PC.DIP).*

*“Teachers have had to face problems for which they had not been prepared” (QO.012.W.1.TON.PC.STE).*

*“We have made an effort to adapt educational programs and aspects such as: content, activities, methodology... but it has not been easy because we do not have experience in networking, hence it will be important to continue training on these issues in the future” (FG. 002.M.2. TON.PC.AMD).*

*“Families and students have been supported to face the new tasks, but the training of families and students is not always adequate” (FG.002.M.2.TON.PC.PIP).*

*“Educational programs have been adjusted. The new learning modes have been reduced, concentrated, and defined with tasks adapted to satisfy the options and challenges faced by students, but we cannot be completely sure of the effectiveness of our approaches” (FG.004.TON.PC.PIP).*

Used tools (UT):

*“[…] We adapt everything. We select some and transform others taken from data repositories such as platforms, protocols. We use methods and means of other public administrations” (FG.004.W.2.TON.UT.DP).*

*“Teachers’ innovative ideas were supported; direct communication was improved using platforms, not just emails. Weekly mini-meetings were held to evaluate the actions taken by everyone” (QO.087.W.1.TON.UT.DP).*

*“In our center, we have used data repositories with interactive resources” (QO.034.M.1.TON.UT.WP).*

*“We have had additional sessions on the use of the platform from a humanistic point of view” (FG.002.W.2.UT.TON.UT.TE). *

*“Real listening and empathy for students facing difficult situations during the pandemic was also improved” (FG.015.W.2.TON.UT.GC).*

*“Among other resources, we have used flipped learning; we have also used online simulations or collaborative tasks on a platform” (QO.065.W.1.TON.UT.TE).*

*“Individual online work and team projects (using institutional platforms) have been encouraged to foster critical judgment (case analysis), along with others such as infographic presentations or workload platforms” (FG.002.W.2.TON.UT.DL).*

*“[…] Work has been done on the transversality of subjects and knowledge. Nuclear and global contents have been integrated in different subjects such as botany or chemistry, for example” (FG.003.M.2.TON.UT.DI.).*

*“We promote the use of digital books and other ICT media to guide and help communication between all participants in our educational community […], informative websites for families, online tools, apps, satisfaction survey forms for families, students, teachers, webinars, videoconferences (Zoom, Meet), applications like WhatsApp […]” (FG.004.TON.UT.WP).*

*Google classroom, Meet […] and we intensify creativity and autonomy with the use of such platforms, networks, and mini videos […]” (FG.004.TON.UT.DP).*

Action development (AD):

*“We designed a comprehensive communication plan for the entire educational community with weekly decisions made after communicating with families, although at times we have detected repeated information or excess information” (QO.023.TON.AD.NV).*

*“We have developed creative proposals for students and families to progress in digital competence” (FG.001.M.2.TON.AD.CT).*

*“At my school, what was improved was the skills of the teachers to help us adapt to online teaching methods and environments. We were given access to all the means we needed but there was no time to train ourselves in all the tools” (QO.123.W.1.TON.AD.NV).*

*“The Department of Educational Innovation has played a key role. Support and motivation have also been essential to improve planning and its adaptation to each student in a flexible and rational way” (QO.033.W.1.TON.AD.AI).*

*“We generate new bases and methods to adapt resources to the needs of students, using different tools and we stimulate cooperative work and student commitment“ (FG.002.TON.AD.NV).*

*“[...] all planning leaders... we have weekly meetings with the school principals (teacher coordinators) and we provide all necessary modifications, such as student tests, assignments, class schedules, and even the breaks, you know [...]” (FG.004.W.2.TON.AD.PT).*

*“[…] Teachers have investigated innovative pedagogical experiences to adapt them to other particular contexts” (FG.002.M.3.TON.AD.TM).*

*“The teachers have given support and empathy to the students to face their tasks with technological tools, but we were not always sufficiently trained in them” (FG.001.M.2.TON.AD.PT).*

*“The work has been personalized as far as possible to meet the learning profile of each student. We have tried to work on co-responsibility, self-development, and commitment, but we should train ourselves to a greater extent on these issues” (FG.001.W.2.TON.AD.TM).*

*“The training activities have been diversified and adapted. Many learning proposals have emerged to meet the particular needs of each student, although it is difficult to know the real results. New ways of teaching and learning have been discovered” (FG.002.M.3.TON.AD.PT).*

*“The pandemic has forced us to reprogram activities and content so that students integrate key learning competencies. We believe this way of acting has benefited students to develop autonomous learning skills” (QO.013.W.1.TON.AD.PT).*

*“[…] The activities were oriented differently. More creative ways of learning were created to engage students and help them discover or create their own solutions” (QO.029.W.1.TON.AD.CT).*

Needs detected (ND):

*“The health control programs in the classrooms were very detailed but there were no methodological guides on the educational processes to be carried out efficiently; many teachers had to work alone, proposing our own proposals” (FG.003.W.2.TON.ND.CCA).*

*“It has not been easy for teachers to motivate students and make them participate because we ourselves were insecure in the use of digital tools that we handled” (QO.008.W.1.TON.ND.COCO).*

*“The concern of pedagogical leaders for the training and updating of students and families in digital competence was important but the teaching staff was not sufficiently prepared at the beginning and communication problems were generated” (QO.027.W.2.TON.ND.CCT).*

*“[…] Some colleagues have had to face personal problems from COVID-19. In reality, they need to be cared for in a more particular way. They need support, cooperation, and understanding” (QO.07.W.1.TON.ND.IEP).*

*“It is really necessary to adapt and intensify cooperation at all levels. Work together and support each other” (QO.09.W.1.TON.ND.CCP).*

*“[...] decisions must be adapted to each particular case, group and communication between them/their families, and teachers must be intensified; in many cases, contradictory information has been sent and received” (FG.003.M.2.TON.ND.COCO).*

*“We carry out video sessions, adapt subjects, and improve the interaction and development of activities between teachers and students” (FG.002.W.2.TON.ND.CCT).*

Training proposals (TP):

*“Research seminars, learning guides, participation in virtual classrooms, oral presentations have been some of the methodologies and resources used in these months” (FG.004.W.2.TON.TP.DL-GE)*

*“We schedule virtual meetings and exchanges and motivate teachers, students, and families while principals help to gather and create a specific bond within the community” (FG.002.M.3.TON-TP.ET-MO).*

*“[…] There has to be a greater involvement of all the people who dedicate ourselves to Education. Professionals must be prepared for multidisciplinary work if we want to ask students to integrate different learning subjects, because working on a single subject is intellectually and emotionally exhausting for both teachers and students” (FG.002.W.3.TON.TP.ET-MW).*

*“We need to train ourselves in ICT assessment tools: subjects, online tests, uses of platforms…” (FG.004.W.2.TON.TP.DL-GE).*

*“We need seminars and training workshops to answer the questions that arise in the virtual learning environments created by the situation of the pandemic” (FG.002.M.3.TON. TP.DMT-T).*

*“Guidance and individual and group tutoring have been fundamental in this crisis, so these actions should be promoted in the centers in the coming months” (FG.004.M.2.TON.TP.ET-PL).*

*“[…] We believe that the didactic activities contributed positively to the training of students in key competences. They also generated their own cooperative work environments” (FG.002.M.3.TON.TP.ET-LL).*

Finally, the qualitative analysis of answers given by participants in the present research, in terms of the future actions to be provided by pedagogical leaders, in order to foment the integral education of students and the corresponding commitment of teachers and families during crisis periods (e.g., pandemics), demonstrated the following points as fundamental:

- To promote, among teachers, the investigation of pedagogical experiences adaptable to every particular school context;
- To help strengthen the professional identity of teachers, both in early training stages and during their practices, and to provide some acknowledgement of their professional importance in the lives and training of students:
- To provide enough time and space for teachers to work on collaborative projects;
- To improve the self-initiative and innovative ICT activities of all teachers inside the schools;
- To invite external experts, providing a different way of doing things and reinforcing the communication bonds that exist between teachers, directors, families, and students;
- To invite external experts to provide a different way of doing things and strengthen the existing communication links between teachers, principals, families, and students. Integrate continuous training in professional hours;
- To promote multidisciplinary work. Encourage collaboration between the political, business, school, technological, institutional, and community environment, in order to improve the quality of Education.

## 5. Discussion and Conclusions

The set of decisions made by pedagogical leaders in the pandemic process have been improved by improvement of the professional identity, the cooperative culture, and the smart use of ICTs; by setting up different and new forms of interpretation or by changing the existing limitations, teachers can provide new educative opportunities for all.

The fundamental objective of the present research was to show the impact of crisis situations on educative systems and pedagogical leaders. They were more conscious of their professional importance inside society to convert education into an integral training activity and in terms of preparing future generations to overcome uncertain times, such as those provoked by the current pandemic. With the improvement of professional identity, the cooperative culture, the smart use of ICTs, and by setting up different and new forms of interpretation or by changing the existing limitations, teachers can actually provide new educative opportunities for all.

This research demonstrated the high valuation of pedagogical leaders in many adequate decisions before COVID-19, as shown by the results emerging from the qualitative analysis. A high number of didactic decisions were valued positively globally.

Other studies on the subject have also evidenced the high pressure inside educational institutions during the COVID-19 pandemic. Harris and Jones [1] have shown that dimensions such as the educational relationship, social distance, the excessive use of ICT, and the tutorial and support of families are all affected by the situation experienced, which was also evidenced by the results obtained in the present research.

Managers see their responsibilities increase when new confinement measures are introduced in Spain, most particularly when these affect the culture and school institutions or tasks performed [18]. Security affects relationships, mutual respect and confidence, empathetic generated actions, and mutual support, which entails new rules for teachers, students, and family cooperation.

Analysis of the three main factors demonstrated, firstly, that the leadership of teachers and principals in schools ‘is a crucial component for successful teaching and learning’, as far as tasks and challenges are concerned, as has been shown by Lieberman [60] and Lieberman and Miller [61].

During critical periods, such as a pandemic, being a pedagogical leader is quite complex, as underlined by York-Barr and Duke [62] and Harris and Jones [1]. Extra time and space must be given to some essential tasks during the teaching–learning process: innovative activities and practices (0.884); design of hybrid teaching environments (0.880); teamwork and teacher cooperation (0.885); new teaching planning process (0.814); motivating ICT usage in teachers (0.794); promoting the didactic use of ICT (0.772); didactic platform usage (0.784); and webs and apps (0.763). All of this has underlined the concern felt by leaders when performing innovative actions or practices, as they need to be designed within hybrid teaching environments, by a team working together inside of the school. Teachers in the debate groups also pointed in that direction: ‘it concerned, most of all, new experiences, the design of new actions, and a significant performance’.

Many teachers actually demanded more competences: ‘The concern of pedagogical leaders for training and updating students and families in digital competences’ is important, as has been shown by the results published in previous studies by Harris and Jones [1], Harris et al. [29], Hulpia and Devos [30], Diez-Gutiérrez and Galjardo [32], Chick et al. [34], Hodges et al. [35], and Van Barneveld et al. [50].

The first factor synthesized the impact and quality of pedagogical leader performance, as they were able to overcome—with some extra dedication—the most negative aspects of these training process during the COVID-19 period.

The second factor represents student activities and professional development of teachers, which are essential components during the learning–teaching process, as they allow for the empowerment of students. During critical times, teachers adapt to the new social environments, as has been developed by Morrison and Arthur [46], Close and Warnwright [47], Fernández and Saw [63], and Tarinabo (2020) [64]. These authors have proposed a new reflexive framework to design programs, considering uncertainties and technological, social, cultural, and environmental changes [65]. Variables for this factor were tasks using classic resources and ICT (0.810), virtual meeting agenda design (0.797), training meeting planning (0.742), individual training activities (0.455), and online tasks (0.568).

These variables and their corresponding values were also confirmed by the focus groups: ‘It synthesizes the educational objectives all teachers have to design training actions’ and ‘the concern of pedagogical leaders for training and updating students and families in the digital competence is real’.

Student and family training needs to be higher in competences such as digital communication and professional identity, as confirmed by studies published by Sinha and Hanuscin [17]; Richter, Brunner, and Richter [24]; and Brown, Macgregor, and Flood [41]. Our lives have become digitized and digital, which requires a properly trained citizenry to face the challenges and novelties that have arisen [66].

The group of pedagogical leader competences (third factor) to be attained by teachers and principals was quite coherent and compact, as has also been shown in the works by Labell and Jacquin [2]; Proguin and Perrenoud [8]; Garant and Letor [9]; Day (2017) [10]; Richter, Brunner, and Richter [24]; Fernández and Shaw [63]; Robbins and Davidhizar [67]; Yukl [68]; and Domínguez et al. [69]. These underline the empowerment needed by leaders to face very complex situations. This factor is comprised of eight competences, including organization (0.959, the most important weight of all), planning (0.941), resource optimization (0.766), management (0.745), making complex decisions (0.716), and responsibility (0.629).

These values were also confirmed by the focus groups. New situations emerged with enhanced communication, assertive and emotional harmony, and empathy and understanding: ‘support was given to teachers’ innovative ideas; direct communication was enhanced using platforms, not only e-mails. There was real listening and empathy towards students facing complicated situations during the pandemic’. The didactic use of technology was enhanced. Educational policies and didactic and pedagogical trends have increasingly advocated for the use of technology inside and outside the classroom as a support mechanism for teaching and learning [70].

Leaders have needed to acquire new and updated competencies, which has helped in setting a new professional identity, as well as a more intense and cooperative dialogue, with a particular satisfaction shown by all professionals during the performance of their activity and during the design of new didactic materials [24,36,41].

The objectives of the present research were attained. The results provided evidence that the decisions made by leaders to improve the training practices and teaching–learning process had an impact on the integral education of students during the vast COVID-19 period. The educative practices were characterized by responsibility, cooperation, and distributed work, with team-based implications for teachers and a positive attitude of families and students within a cooperative and interactive empathic environment.

Teachers participating in the study gave high values to the tasks performed by the pedagogical leaders that improved the teaching–learning process during the pandemic. The performance of tasks and its significance was reflected in the most characteristic experiences recorded in the focus groups: ‘we designed an integral communication planning for the whole community and performed weekly meetings with exchanges and actions that corresponded to family demands’, ‘we did video classes, adapted actions, and provided time for families using different ICT tools (webs, webinars, Zoom, etc.)’.

The adaptation of the school programs was due to the essential need to change agendas and subjects or thematic issues, as shown by the values $\overset{-}{X}$ = 5.49 and $\overset{-}{X}$ = 5.63, respectively (see Appendix A). As reflected by focus groups: ‘we adjusted programs and redefined new task models focused on students’ challenges and options’, ‘we worked more on a personalized way to respect every student’ s profile and respected the co-responsibility and personal self-development of all’.

The interests of the leaders in the focus groups confirmed the need to complement the individual tasks with some group ones, to stimulate the cooperation between families, and to broaden the training activities provided by teachers: not only those using ICTs, but also seminars and innovative teaching actions. The Planning and Training dimensions obtained high values ($\overset{-}{X}$ = 5.06 and 5.01, respectively) and, though one of the training activities obtained a minor value ($\overset{-}{X}$ = 4.37), globally harmonized results were observed. This provides an integral gathering of the re-planning process and teachers‘ training and training activities quality dimensions, which are fundamental to advance teachers’ competencies actualization, as showed by other research such as Harris and Jones [1], Thornton [25], and Chick et al. [33].

The incidence of this new didactic interaction was marked by empathy, closeness, and a social climate created by pedagogical leaders, students, and families, as evidenced by Darling-Hammond and Cook-Harvey (2018) [18] and Medina et al. [48]. The results indicated the huge educative impact of the interaction between all members of the community, as underlined by Touriñán [71]; the educational interaction is potentiated to facilitate quality-based education. ICT resources have eased the educative interaction, and teachers have underlined they have had the opportunity to get closer to families and students. This has helped to minimize the isolation experienced and facilitated one-to-one tutorials by focusing on emotions and relationships.

Among the highest valued competences when facing the challenging COVID-19 issues are human-based competencies, as has already been shown in previous work (Medina and Gómez, 2014). These included responsibility ($\overset{-}{X}$ = 5.65) and empathy ($\overset{-}{X}$ = 5.64), together with making decisions in complex times ($\overset{-}{X}$ = 5.47). These competencies are assumed, organized, and provide for the most pertinent qualities to be creative and rigorous when facing uncertain situations. The most relevant competencies to overcome isolation situations and excessive dependence on ICTs are communication and emotional empathy, as shown in the previous works of Day [11], Domínguez-Garrido et al. [72], and Medina and Pérez [73].

## 6. Research Limitations

The process of applying the instruments applied in this research was lengthy and affected by the uncertain and complex situations experienced by the pedagogical leaders themselves. Consequently, we attempted to overcome the double limitation of time dedicated to the leaders to answer the instruments presented and to present them in the discussion groups, as well as—in some cases—the breadth of some of the open questions in the questionnaire. We hope to overcome these limitations in future research.

The sample was limited, given the triple incidence of the pandemic process itself, the complexity experienced by teachers in the face of new distance education processes, and the intense management of ICT, which has represented an added effort for teachers, families, and students. We considered the sample to be relevant and sufficient, although the level of representativeness was limited, and the type of sampling was intentional and with the support of supervisors who encouraged response to the instruments, participation in the discussion groups, and the generosity shown in the answers given to open questions.

## Figures and Tables

**Table 1:** Best leadership practices.

Leadership Practices in Normal Times	Leadership Roles in Times of Crisis
To model the way (Finding one’s voice)	Sense-maker (Reflection and creativity-based decisions)
To inspire the way (To assert values, to imagine a possible future)	Technology enabler (To wisely integrate and use ICTs)
To process challenges (To assume risks)	Emotional stability and applied well-being (To make emotionally balanced decisions and implement them among the staff)
To evaluate actions performed by others. (Creating a climate for evaluation)	Innovative communication (To perform fluid and strict communication by different means)

**Table 2:** Acronyms and explanation for the coding of the participants in terms of the qualitative information collection techniques used for this study.

Acronym	Explanations
	*Provides information about the origin of the meaning segment*
QO	QO: Question Open
FG	FG: Focus Group
Number	*Digits to identify the document (interview or discussion group)*
	(001, 002, 003, …)
	*Identify gender*
M	M: Man
W	W: Woman
	*Adjusts the age frame*
1	1: from 22 to 40 years old
2	2: from 41 to 54 years old
3	3: older than 55
	*Level of studies*
SG	SG: School Graduate
VT	VT: Vocational Training
US	US: University Studies
D	D: Doctor
	*Profile in the study*
DI	DI: Direction
CO	CO: Coordination
TE	TE: Teacher
ST	ST: Student
Example	In focus group number 1, a man responds who is between 41 and 54 years old, with a university education level and who currently performs institutional management and leadership tasks. Coding: (FG.001.M.2.UE.DI.)

**Table 3:** Means obtained by questionnaire dimensions.

Dimension	Mean
II. Digital competencies training (students and families)	5.09
III. Communication Planning	4.97
IV. Educational program adaptation	5.20
V. ICT resources and places of use	5.19
VI. Teaching and learning process planning and development	5.06
VII. Training activities	4.57
VIII. Educational interaction	5.02
IX. Teacher training	5.01
X. Pedagogical leader competence in pandemic times	5.44

**Table 4:** Measures of factor adequacy.

KERRYPNX	F1	F2	F3
SS loadings	16.46	9.65	7.25
Proportion Var.	0.32	0.19	0.14
Cumulative Var.	0.32	0.51	0.65
Proportion Explained	0.49	0.78	1.00
Cumulative proportion entry	0.49	0.78	1.00

**Table 5:** Correlation between factors.

	F1	F2	F3
F1	1.00	0.42	0.41
F2	0.42	1.00	0.35
F3	0.41	0.35	1.00

**Table 6:** Results of factorial analysis: F1–F2.

	Items	F1	F2
F1	05. Promoting student training	0.517	
	06. To support student activities	0.477	
	07. To enhance all Administration actions	0.585	
	08. To stimulate ICT, use by teachers	0.490	
	13. To motivate ICT, use in teachers	0.794	
	14. Communication platforms	0.702	
	15. Webs/Apps	0.763	
	16. Social networks	0.500	
	17. Mini-videos	0.706	
	18. Webconferences	0.565	
	21. Ed. Program adaptation	0.731	
	22. Innovative activities and practices	0.884	
	23. Diversity-based resources	0.658	
	24. Teamwork and teacher cooperation	0.855	
	25. Student Tutorials	0.687	
	27. To assess and promote the didactic use of ICT	0.772	
	28. Provision of easily accessible platforms	0.716	
	29. Didactic platforms Usage	0.784	
	30. Design of hybrid teaching environments	0.880	
	31. Smart use of mobile phones	0.734	
	32. Didactic making of mini videos.	0.750	
	33. ICT resources demands to Administrations	0.504	
	35. New teaching Planning process	0.814	
	36. Ed. Program re-orientation and planning	0.688	
	37. Adapting program criterial	0.734	
	38. To ease student task selection	0.718	
	51. To enhance student interactions	0.684	
F2	42. Individual training activities		0.455
	43. On-line tasks		0.568
	44. Tasks using classic resources and ICTs		0.810
	54. Teacher training in ICT use		0.675
	55. Design of a virtual meeting agenda		0.797
	56. Training meeting Planning		0.742
	57. To plan seminars and workshops		0.689
	58. To present innovative training models		0.639

**Table 7:** Results of Factorial analysis: F3.

	Items	F3
F3	65. Responsibility	0.629
	66. Patience	0.608
	68. Management	0.745
	69. Planning	0.941
	70. Organization	0.959
	71. Resources Optimization	0.766
	72. Taking complex decisions	0.716
	74. Technical and pedagogic or didactic	0.552

**Table 8:** Results that emerged in the segments of meaning obtained in the interviews and discussion groups for the qualitative analysis (carried out with Aquad 8).

Catalogue	Categories	Code	f **	F ***	FT ****
Perceived Context (PC)	Coherent Health Protocols (CHP)	TON-PC-CHP	08	047	578
	Individual Technology Initiatives (ITI)	TON-PC-ITI	09		
	Political Insufficient Protocols (PIP)	TON-PC-PIP	07		
	Insufficient Administrative Protocols (PAP)	TON-PC-PAP	03		
	Dependent Institutional Protocols (DIP)	TON-PC-DIP	10		
	Absence of Methodological Guidelines (AMD)	TON-PC-AMD	11		
	Mismatched Educational Resources (MER)	TON-PC-MER	09		
	Redundant Communication (RCO)	TON-PC-RCO	06		
	Obsolete Legal Framework (OLF)	TON-PC-OLF	04		
	Soledad Teacher (STE)	TON-PC-STE	10		
Used Tools (UT)	Distribution Lists (DL)	TON-UT-DL	10	101	
	Google Meets (GM)	TON-UT-GM	09		
	Google Classroom (GC)	TON-UT-GC	09		
	Wasp (WA)	TON-UT-WA	11		
	Digital Books (DB)	TON-UT-DB	07		
	Mobile Phone (MP)	TON-UT-MP	10		
	Email (EM)	TON-UT-EM	12		
	Internet (IN)	TON-UT-IN	13		
	Teams (TE)	TON-UT-TE	07		
	Web Pages (WP)	TON-UT-WP	13		
Actions Developed (AD)	Read Information (RI)	TON-AD-RI	14	102	
	Search Information (SI)	TON-AD-RI	12		
	Analyze Information (AI)	TON-AD-AI	10		
	Exchange Information (EI)	TON-AD-EI	10		
	Complete Information (CI)	TON-AD-CI	12		
	Troubleshoot (TP)	TON-AD-TP	09		
	Creative Tasks (CT)	TON-AD-CT	05		
	Teamwork (TE)	TON-AD-TE	04		
	Networking (NW)	TON-AD-NW	03		
	Digital Training (DT)	TON-AD-DT	04		
	Plan Teaching (PT)	TON-AD-PT	02		
	Teaching Methodologies (TM)	TON-AD-TM	03		
	Student Assessment (SA)	TON-AD-SA	01		
	Administrative Management-Network (AM)	TON-AD-AM	10		
	Educational Management-Network (EM)	TON-AD-EM	03		
Needs Detected (ND)	Cultural Change Teaching (CCT)	TON-ND-CCT	12	198	
	Cultural Change Directorate (CCD)	TON-ND-CCD	10		
	Cultural Change Administration (CCA)	TON-ND-CCA	08		
	Political Cultural Change (PCC)	TON-ND-PCC	08		
	Collective Coordination (COCO)	TON-ND-COCO	14		
	Political Involvement Education (PIE)	TON-ND-PIE	12		
	Institutional Involvement Education (PIED)	TON-ND-PIED	11		
	Involvement Administration Education (IAE)	TON-ND-IAE	10		
	Business Education Involvement (BEI)	TON-ND-BEI	03		
	Effective Communication Management (ECM)	TON-ND-ECM	08		
	Improvement of Educational Processes (IEP)	TON-ND-IEP	10		
	Digital Materials Management (DMM)	TON-ND-DMM	06		
	Effective Digital Platforms (EDP)	TON-ND-EDP	05		
	Adequacy of the Regulations (AR)	TON-ND-AR	04		
	Competency: Learn to Learn (CLL)	TON-ND-CLL	10		
	Competence: Curricular Adaptation (CCA)	TON-ND-CCA	07		
	Competence: Digital Assessment (CDA)	TON-ND-CDA	09		
	Competence: Planning (CPL)	TON-ND-CPL	07		
	Competence: Leadership (CLI)	TON-ND-CLI	06		
	Resilience Capability (RECA)	TON-ND-RECA	05		
	Capacity for Empathy (EMCA)	TON-ND-EMCA	04		
	Motivational Capability (MOCA)	TON-ND-MOCA	09		
Training Proposals (TP)	Digital Literacy: Generalized (DL-GE)	TON-TP-DL-GE	15	130	
	Digital Management Training: Management (DMT-M)	TON-TP-DMT-M	10		
	Digital Management Training: Teachers (DMT-T)	TON-TP-DMT-T	11		
	Educational Training: Communication (ET-C)	TON-TP-ET-C	10		
	Educational Training: Leadership (ET-LI)	TON-TP-ET-LI	07		
	Digital Educational Training: Teachers (DET-DO)	TON-TP-DET-DO	08		
	Digital Educational Training: Students (DET-A)	TON-TP-DET-A	10		
	Digital Educational Training: Families (DET-F)	TON-TP-DET-F	07		
	Educational Training: Resilience (ET-R)	TON-TP-ET-R	05		
	Educational Training: Empathy (ET-EM)	TON-TP-ET-M	04		
	Educational Training: Motivation (ET-MO)	TON-TP-ET-MO	06		
	Educational Training: Multidisciplinary Work (ET-MW)	TON-TP-ET-MW	08		
	Educational Training: Learn to Learn (ET-LL)	TON-TP-ET-LL	10		
	Educational Training: Learning to Entrepreneurship (ET-LE)	TON-TP-ET-LL	06		
	Educational Training: Planning (ET-PL)	TON-TP-ET-PL	08		
	Educational Training: Evaluation (ET-EV)	TON-TP-ET-EV	10		

Notes: f**, frequency; F***, relative frequency; FT****, total frequency.

## Data Availability

The data presented in this study are available on request from the corresponding author. The data are not publicly available due to confidentiality agreements with participants.

## Appendix A

**Table A1:** Descriptive analysis on questionnaire items.

Dimension	Items	M	SD	V
II	5. Promoting student training	5.29	0.74	0.55
	6. To support student activities	5.22	0.88	0.77
	7. To enhance all Administration actions	5.19	0.91	0.84
	8. To stimulate ICT use by teachers	5.34	0.74	0.55
	9. To train family members	4.45	1.34	1.79
III	11. Communication planning	4.63	1.52	2.32
	12. Student assessment	4.65	1.46	2.14
	13. To motivate ICT use in teachers	5.33	0.98	0.97
	14. Communication platforms	5.38	0.90	0.81
	15. Webs/Apps	5.17	1.05	1.11
	16. Social medias	4.50	1.51	2.29
	17. Mini-videos	4.87	1.26	1.59
	18. Webconferences	5.24	0.90	0.81
IV	21. Ed. Program adaptations	5.27	0.96	0.92
	22. Innovative activities and practices	4.98	1.15	1.33
	23. Diversity-based resources	5.23	1.09	1.19
	24. Teamwork and teacher cooperation	5.47	0.75	0.57
	25. Student tutorial	5.07	1.03	1.08
V	27. To assess and promote the didactic use of ICTs	5.28	0.90	0.82
	28. To provide easily accessible platforms	5.39	0.77	0.60
	29. Didactic platform usage	5.44	0.83	0.70
	30. Design of hybrid teaching environments	5.13	0.95	0.90
	31. Smart use of mobile phones	4.87	1.25	1.57
	32. Didactic making of mini videos	4.90	1.16	1.35
	33. ICT resources demands to Administrations	5.35	0.91	0.83
VI	35. New teaching planning process	5.18	0.091	0.83
	36. Ed. Program re-orientation and planning	5.20	0.899	0.80
	37. Adaptation of program criteria	5.32	0.751	0.56
	38. To ease student task selection	5.04	1.11	1.24
	39. Tasks adapted to specific needs of students	4.59	1.28	1.64
VII	41. To elaborate jointly the task agenda	4.45	1.24	1.54
	42. Individual training activities	4.55	1.25	1.56
	43. On-line tasks	4.45	1.36	1.86
	44. Tasks using classic resources and ICTs	4.83	1.20	1.45
VIII	49. To harmonize all interactions	5.26	0.84	0.70
	50. To adapt actions to number of students attending	4.83	1.11	1.24
	51. To impulse student doubts resolution actions	4.99	1.06	1.12
IX	54. Teacher training in learning environments	4.95	1.10	1.21
	55. Design of virtual/physical meeting agenda	5.06	1.10	1.22
	56. Planning of training meetings	5.00	1.12	1.26
	57. To impulse seminars and workshops	5.06	1.03	1.07
	58. To present innovative training models	5.01	1.05	1.10
X	61. Human values	5.71	0.69	0.48
	62. Empathy	5.64	0.62	0.38
	63. Emotional	5.46	0.92	0.85
	64. Models to follow	5.31	1.11	1.24
	65. Responsibility	5.65	0.63	0.40
	66. Patience	5.66	0.74	0.55
	68. Management	5.43	0.84	0.72
	69. Planning	5.47	0.86	0.74
	70. Organization	5.50	0.83	0.70
	71. Resources optimization	5.40	0.90	0.82
	72. Taking complex decisions	5.47	0.900	0.81
	74. Technical and pedagogic or didactic	5.21	1.02	1.05
	75. Planning Institutional Projects	4.89	1.17	1.38
